# Closing the gap in implementation of HIV clinical guidelines in a low resource setting using electronic medical records

**DOI:** 10.1186/s12913-020-05613-8

**Published:** 2020-08-26

**Authors:** Adrien Allorant, Canada Parrish, Gracia Desforges, Ermane Robin, Jean Guy Honore, Nancy Puttkammer

**Affiliations:** 1 Institute for Health Metrics and Evaluation, Department of Global Health, University of Washington, 2301 5th Avenue, Seattle, WA 98121 USA; 2 Department of Health Services Research, UW, Seattle, WA USA; 3 Ministère de la Santé Publique et de la Population (MSPP), Port-au-Prince, Haiti; 4 Centre Haïtien pour le Renforcement du Système de Santé (CHARESS), Port-au-Prince, Haiti; 5 Department of Global Health, International Training and Education Center for Health (I-TECH), UW, Seattle, WA USA

**Keywords:** Quality of care, Healthcare delivery, Electronic medical record, LMIC, HIV, Outliers, Funnel plots

## Abstract

**Background:**

Universal health coverage promises equity in access to and quality of health services. However, there is variability in the quality of the care (QoC) delivered at health facilities in low and middle-income countries (LMICs). Detecting gaps in implementation of clinical guidelines is key to prioritizing the efforts to improve quality of care. The aim of this study was to present statistical methods that maximize the use of existing electronic medical records (EMR) to monitor compliance with evidence-based care guidelines in LMICs.

**Methods:**

We used *iSanté,* Haiti’s largest EMR to assess adherence to treatment guidelines and retention on treatment of HIV patients across Haitian HIV care facilities. We selected three processes of care – (1) implementation of a ‘test and start’ approach to antiretroviral therapy (ART), (2) implementation of HIV viral load testing, and (3) uptake of multi-month scripting for ART, and three continuity of care indicators – (4) timely ART pick-up, (5) 6-month ART retention of pregnant women and (6) 6-month ART retention of non-pregnant adults. We estimated these six indicators using a model-based approach to account for their volatility and measurement error. We added a case-mix adjustment for continuity of care indicators to account for the effect of factors other than medical care (biological, socio-economic). We combined the six indicators in a composite measure of appropriate care based on adherence to treatment guidelines.

**Results:**

We analyzed data from 65,472 patients seen in 89 health facilities between June 2016 and March 2018. Adoption of treatment guidelines differed greatly between facilities; several facilities displayed 100% compliance failure, suggesting implementation issues. Risk-adjusted continuity of care indicators showed less variability, although several facilities had patient retention rates that deviated significantly from the national average. Based on the composite measure, we identified two facilities with consistently poor performance and two star performers.

**Conclusions:**

Our work demonstrates the potential of EMRs to detect gaps in appropriate care processes, and thereby to guide quality improvement efforts. Closing quality gaps will be pivotal in achieving equitable access to quality care in LMICs.

## Background

The recent report from The Lancet Global Health Commission on High Quality Health Systems (HQSS) highlighted the importance of improving quality of care (QoC) in order to reach the Sustainable Development Goals (SDGs) in health [1]. While measurement is key to progress and accountability, assessing QoC, especially in low and middle-income countries (LMICs) where data are often scarce, is challenging. The fundamental problem of quality assessment resides in the fact that medical care is multifaceted. In his seminal work on QoC, Donabedian distinguishes three main approaches to quality assessment: studies focusing on structures, processes, and outcomes [2]. While structures condition an environment “conducive or inimical to the provision of good care”, and outcomes reflect changes in health status attributable to multiple biological and socio-economic factors as well as antecedent care, processes of care are a direct measure of health providers’ practices, or “technical performance” [3]. Therefore, Donabedian argues that processes with documented benefits on desired outcomes –commonly referred to as evidence-based care – constitute the preferred indicators of quality, when available. Evidence-based guidelines are a central component of the delivery of appropriate care, along with clinical expertise, patient-centeredness, resource use, and equity [4]. Inappropriate care includes the underuse of effective care and the overuse of unnecessary care. Examples of inappropriate care in LMICs highlighted in the HQSS report encompass the omission of oral rehydration therapy and the unnecessary use of antibiotics to treat children with diarrhea, which can result in child death and antimicrobial resistance, and the low uptake of HIV antiretroviral therapy, despite the effectiveness of the treatment in reducing deaths and suffering from HIV/AIDS. Overall, health providers in LMICs fulfill less than 50% of recommended clinical guidelines, on average [1].

Medical records constitute the prime data source to assess adherence to evidence-based clinical guidelines [5]. In high-income countries, electronic medical records (EMRs) are widely used to monitor clinical practices and to study regional variation of medical practices in the US [6]. In LMICs, EMRs have become more prevalent; as of 2015, 34 countries had adopted a national EMR [7], and 67 were currently using the District Health Information System 2 [8]. However, EMRs are often overlooked to study QoC in LMICs, and large community surveys, such as the Demographic and Health Surveys (DHS) Service Provision Assessments, remain the primary choice for quality assessments [9]. The main motivation to favor large surveys over EMRs is the alleged better representativeness, completeness, and timeliness of the former [10]. As discussed by Wagenaar and colleagues, the superiority of large surveys is however debatable and varies over locations and time [11]. Furthermore, the data quality of EMRs has greatly benefitted from investments in data management, and the development of internal data check algorithms [12]. Besides, EMRs are routinely used as the primary data source for informing resource allocations in performance-based financing programs [13, 14]. In this paper, we argue that national EMRs have the potential to drive country-led quality measurement and improvement in LMICs.

We worked with Haiti’s Ministry of Health and Population, and Haiti’s Center for Health System Strengthening, on the analysis of Haiti’s largest EMR system, i*Santé* [15]. We utilized i*Santé* data on patients’ demographics, medical history, progress and care received, and applied robust statistical methods to detect outlying performance in the clinical activities provided in Haiti’s HIV facilities. Typical methods for performance monitoring, such as scorecard measurements, consist in comparing observed indicators with pre-specified targets to determine achievement, underachievement or failure [16]. These methods however fail to account for sampling variability when dealing with small sample size or volatile indicators, and do not allow to adjust for risk-varying patient profiles [17–19]. The methods presented in this paper account for these shortcomings, while being straightforward enough to be explained to multiple stakeholders, and could be easily implemented as an additional automated layer on a health information system [20]. The aim of these statistical methods is not to issue a comprehensive judgement on the QoC dispensed in facilities, including structural aspects of QoC, but rather to extract from a complex operational information system a signal suggesting outlying variation in appropriate care [21]. In countries with limited financial resources and staffing to support quality management and oversight through site visits, a risk-based approach to drive more targeted facility inspections can help allocate resources where they are the most needed. The aim of this study is to present robust statistical methods that optimize an existing EMR to monitor appropriate care in compliance with HIV care and treatment guidelines in Haitian facilities. These methods could be replicated in conjunction with other large-scale networked EMRs, extending the focus to common HIV co-morbidities such as malaria [15] or to other areas of primary care such as maternal and child health. Therefore, one objective of the present work is to act both as a proof of concept and as an incentive for countries to maximize the use of their health information systems for country-led quality measurement and improvement.

## Methods

### Data source and variables

We utilized clinical, pharmacy, and laboratory data from i*Santé*. Haiti’s largest EMR system is operated by the Ministry of Public Health and Population, and includes data from approximately 70% of all patients on antiretroviral therapy (ART) in Haiti [15]. This secondary analysis involved data from 65,472 patients in 90 health facilities from June 2016 to March 2018.

### Measures of QoC

With the spread of ART, most LMICs with a high HIV burden like Haiti face the challenge of diagnosing, initiating and retaining patients on treatment in order to meet the targets for HIV epidemic control embraced by WHO and PEPFAR [22].

We considered three processes of care and three continuity of care indicators to construct a composite measure of appropriate care at the health-facility level (**see** Table 1). Processes of care refer to clinical activities and our indicators encompassed (1) the implementation of a universal ‘test and start’ approach to initiating ART, (2) the implementation of HIV viral load testing, and (3) the uptake of multi-month scripting (MMS) for stable ART patients. We chose to monitor these three processes of care as they are crucial to initiate (1) and maintain (2) effective individual ART, and a core component of Haiti’s differentiated care approach (3). We used the term continuity of care indicators to refer to indicators measuring the attainment of a specific clinical objective. Our three indicators were chosen to reflect facilities’ ability to retain patients on ART. The first indicator was timely pick up of ART medications among all ART patients, a marker related to long-term retention on ART [23, 24]. The following two indicators were retention 6 months after ART initiation for new ART patients, assessed separately for pregnant and non-pregnant adults, as the features of caring for HIV pregnant women are arguably rather specific [25, 26].

**Table 1:** Definition of the six quality of care indicators

Process of care indicators	Definition
Universal test and start approach	Patient started ART within 1 month of HIV diagnosis
Uptake of viral load testing	Patient up-to-date with respect to viral load testing
Uptake of Multi-Month Scripting (MMS)	Use of MMS per guidelines based on definition of stable patients who are eligible for MMS
**Continuity of care indicators**	
Timely pick-up of ART	Patient has returned within 30 days of expected ART refill date
ART 6-month retention among non-pregnant adults	Patient has returned within 30 days of expected ART refill date, 6 months after starting ART
ART 6-month retention among pregnant women	Patient has returned within 30 days of expected ART refill date, 6 months after starting ART AND patient pregnant or postpartum at time of starting ART

### Models

#### Process of care indicators

We defined each of the three process indicators as the proportion of patients in each facility who received care that was out of compliance with standards: (1) the proportion of patients who were not initiated on treatment within the month of their HIV diagnosis; (2) the proportion of patients who were not up-to-date with their viral load test; and (3) the proportion of patients inappropriately assigned a MMS prescription.

#### Continuity of care indicators

Continuity of care measures are subject to confounding when comparing performances of facilities with different patient mix [27, 28]. Timely pick up of ART medications or retention at 6 months can arguably be affected by other factors than QoC, such as distance to the HIV facility. Therefore, our analysis of retention included a case-mix adjustment, which included demographics and baseline clinical covariates (**see** Table 2), to attribute to QoC the observed differences in indicators between facilities. We conducted a binomial (indicator 4) and two logistic regression models (indicators 5 and 6) to obtain individuals’ expected probability of timely ART pick up, and of being retained on treatment 6 months after initiation of ART, based on demographics and baseline clinical covariates. From these probabilities, we derived the expected proportion of late ART pick up, and the expected proportion of patients not retained on ART for each facility. The case-mix adjustment consisted of subtracting the observed proportion from the expected proportion.

**Table 2:** Covariates used for the case-mix adjustment

Patient characteristics	Variable	Category	N (%)
	Age at HIV diagnosis (mean (sd))		36.7 (13.3)
	Gender		
		Male	23,723 (36.4)
		Female	41,505 (63.5)
		Missing	241 (< 0.01)
	Marital status		
		0	32,961 (50.3)
		1	8534 (13.0)
		2	11,914 (18.2)
		3	12,060 (18.4)
	Pregnancy or post-partum adults at ART initiation		
		Yes	19,061 (29.1)
		No	46,408 (70.9)
	Body mass index at ART initiation (mean (sd))		22.1 (4.1)
	WHO stage at ART initiation		
		1	16,104 (28.1)
		2	12,444 (21.7)
		3	12,858 (22.5)
		4	15,845 (27.7)
	Year of ART initiation		
		2004	35 (0.1)
		2005	278 (0.4)
		2006	728 (1.1)
		2007	1256 (1.9)
		2008	2047 (3.1)
		2009	2146 (3.3)
		2010	2021 (3.1)
		2011	3236 (4.9)
		2012	4855 (7.4)
		2013	6071 (9.3)
		2014	7314 (11.2)
		2015	7117 (10.9)
		2016	11,273 (17.2)
		2017	14,108 (21.5)
		2018	2984 (4.6)
**Facility characteristics**			
	Average travel time to facility (mean (sd))		67.6 (67.3)
	Facility’s slope (mean (sd))		3.2 (2.2)
	Facility’s department		
		Ouest	27,218 (41.6)
		Nord	12,576 (19.2)
		Sud	6059 (9.3)
		Artibonite	5889 (9.0)
		Nord Ouest	4218 (6.4)
		Nippes	2589 (4.0)
		Sud Est	2002 (3.1)
		Nord Est	2872 (4.4)
		Grand Anse	2046 (3.1)
	Facility’s category		
		Univ Hosp	4982 (7.6)
		Dep Hosp	27,342 (41.8)
		Other hosp	668 (1.0)
		HCR	13,103 (20.0)
		CAL	18,412 (28.1)
		CSL/disp	962 (1.5)

### Statistical analysis of outlying performance

Each of our six indicators can be seen as a proportion of failure; failure being defined as the non-compliance to the standard care (indicators 1–3), or as patients not retained on treatment (indicators 4–6). We used binomial distributions to model indicators 1–3. For indicators 4–6, we had to examine other distributions to account for the case-mix adjustment. Building on Ohlssen, Sharples, and Spiegelhalter’s work [18] we considered the difference between the logit of the observed and the expected proportions of success, noted *y*_*i*_ for *i = 1, …,90* facilities_,_ and made a normal approximation.

Having specified for each indicator a distribution from which the observations were drawn (binomial for indicators 1–3; normal distribution for indicators 4–6), we could formally define outliers as observations more than two standard deviations lower or higher than the common mean (the national average proportion of failure for indicators 1–3, and zero for indicators 4–6). This corresponds to a test of a distributional hypothesis for each facility; the null hypothesis being “H_0_: the facility is drawn from the overall distribution”. We could deduce a *p-value* for each facility and each indicator*,* i.e. the probability of observing a value at least as extreme as that which was observed for indicator *k = 1, …,6* under H_0_. Facilities with a *p-value* below a critical threshold, typically *α = 0.05*, could be identified as potential outliers. However, as we tested this hypothesis for each facility, several facilities would have been detected as outliers due solely to chance [19]. To avoid multiplicity testing, we used instead a Bonferroni-corrected critical threshold: α/*I*, where *I = 90*, the number of facilities.

#### Funnel plots

**Fig. 1:**
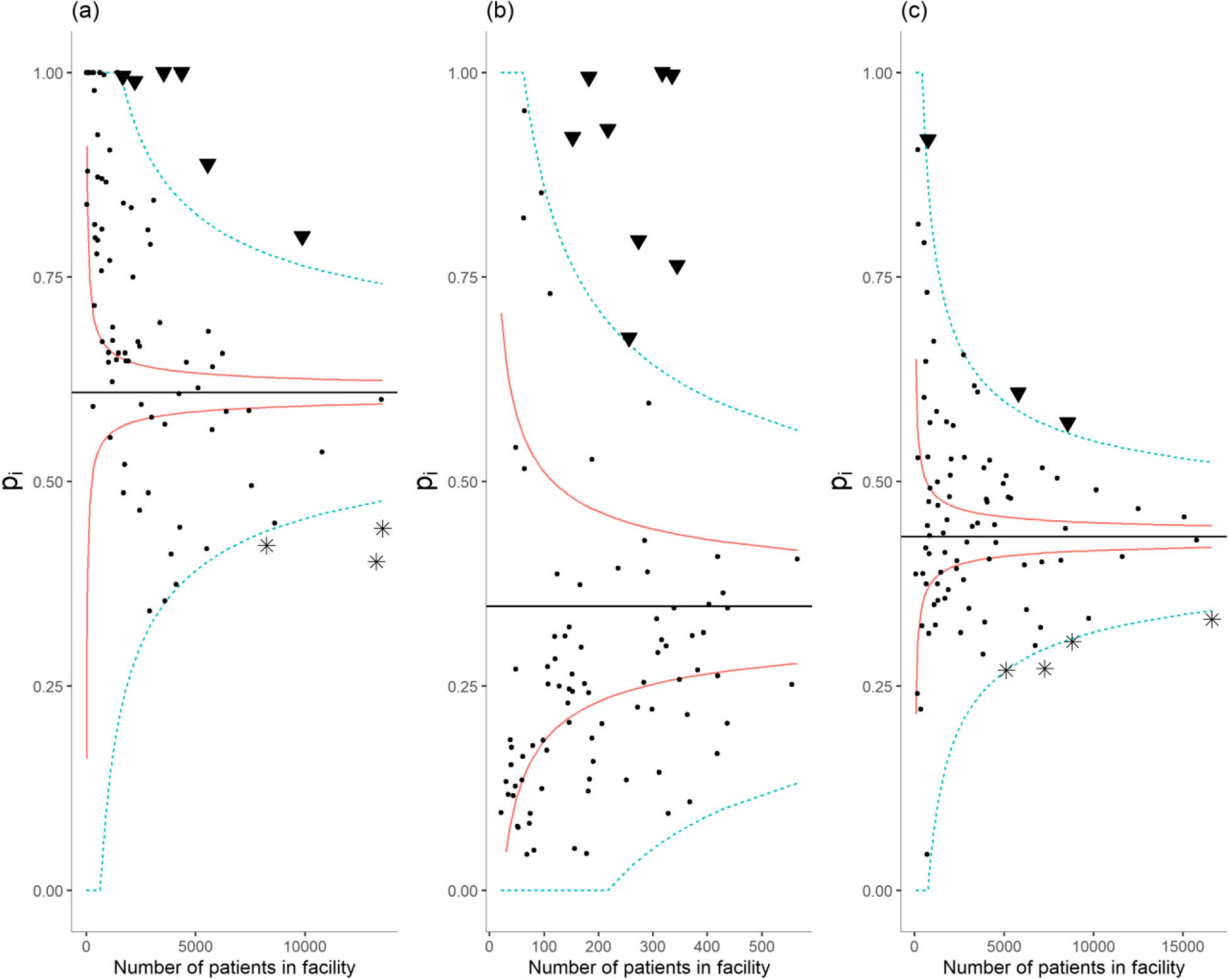
Funnel plots of facility-specific proportion of patients who were not initiated on treatment within the month of their HIV diagnosis (**a**), who were not up-to-date with their viral load test (**b**), who were inappropriately assigned a MMS prescription (**c**) **Sites with typical (●), and outlying low (▼) or high (⁎) performances.**  Bonferroni-corrected 95% confidence interval.  Bonferroni-corrected 95% confidence interval accounting for over-dispersion

**Fig. 2:**
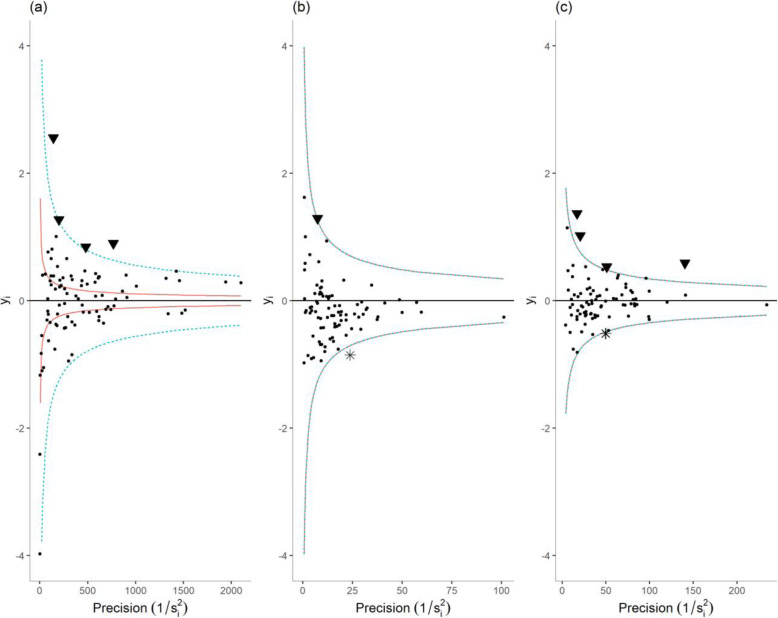
Funnel plots of facility-specific logit ratio of observed versus expected rates: (**a**) of timely ART pick-up; (**b**) of 6-month retention among non-pregnant adults; and (**c**) of 6-month retention among pregnant or post-partum women at ART initiation. Sites with typical (●), and outlying low (▼) or high (⁎) performances.  Bonferroni-corrected 95% confidence interval.  Bonferroni-corrected 95% confidence interval accounting for over-dispersion

Funnel plots have been designed as a visualization tool for comparison of institutional performance [17]. On a funnel plot, each performance indicator is plotted against a measure of its precision. This corresponded to the number of “trials” for indicators 1–3 (Fig. 1), which is the total number of patients (indicators 1–2) or the total number of prescriptions (indicator 3) in each facility. For indicators 4–6 (Fig. 2), we took the inverse of each facility estimate’s (*y*_*i*_) standard error (*s*_*i*_), as the measure of its precision. The horizontal solid line indicates the target, π_0_ the average proportion of failure across all facilities for indicators 1–3, and zero for indicators 4–6. Finally, the solid and dashed lines that form a funnel around the target are the control limits, the graphical counterpart of prediction intervals. The location of the control limits depends on the distribution under H_0_; a binomial distribution with mean π_0_ for indicators 1–3, and a normal distribution with mean zero for indicators 4–6. A second set of control limits were added on each funnel plot; they correspond to another specification of the null distribution accounting for over-dispersion.

#### Over-dispersion

Over-dispersion occurs when the within-facility variability is underestimated. It leads to a low coverage probability of the confidence interval, or equivalently, an inappropriately high number of outliers. Over-dispersion is common when modeling data with a binomial or a Poisson distribution because of the fixed mean-variance relationship [29]. As binomial distributions were involved in modeling either the proportions of failure (indicators 1–3), or the expected proportions of failure given case-mix adjustment (indicators 4–6), we expected that our indicators would display over-dispersion. Therefore, we inflated the model-based standard errors by $\sqrt{\varphi }$, with φ an over-dispersion parameter (see Additional file 1).

In a sensitivity analysis, we used an alternative to the multiplicative over-dispersion model described above: we considered an additive random-effects model, which assumes that each facility has its own true underlying achievement level, normally distributed around the national average.

#### Creation of a composite performance indicator

A z-score represents the deviation from a standard on a common scale. Our six indicators were heterogeneous; we transformed them into z-scores to place them on a common scale (see Additional file 1). Furthermore, we combined the six z-scores to obtain a single composite measure of performance for each facility. We first capped all z-scores to ±3, to avoid one dimension of QoC driving the value of a facility’s composite measure, and then calculated a weighted mean of the six z-scores, to adjust for correlation between indicators. The details of this calculation appear in Additional file 1. Assessing providers’ compliance with core standards in England, Bardsley et al. [20] suggested to weight indicators on a three-point scale based on their relevance and reliability (low (.5), medium (1), high (1.5)) so that they contribute differently to the final composite indicator used to target local inspection. Similarly, we introduced relevance weights, determined via a qualitative process with input from HIV program stakeholders in Haiti, to reflect the perceived data quality of each indicator and its relevance to a composite indicator measuring facilities’ level of appropriate care. Indicators 1 to 6 were assigned the following relevance weights: 1.5, 1.5, 1.5, 0.5, 0.5, and 1. To assess the impact of these weights on the results, we performed a sensitivity analysis where the six indicators were equally weighted.

The composite z-score can be seen as our overall measure of quality of care for each facility. We calculated it for 89 out of the 90 facilities, as one facility had no patient eligible for three out of the six indicators.

## Results

### Process of care indicators

Figure 1 shows funnel plots for each process of care indicators. An overall comment is that all three indicators displayed evidence of over-dispersion; we see indeed an inappropriately high number of facilities lying outside the solid lines. The wider funnels accounting for over-dispersion seems more appropriate to label facilities as significant outliers. Eight facilities fall above this stricter limit for indicator 1, indicating unusually poor performance, while zero fall below (Fig. 1a). In three facilities, the proportion of failure on indicator 1 was 100%, which raises questions about systematic issues preventing these sites from implementing the ‘test and start approach’, such as lack of training on the revised guidelines. Figure 1b shows that the average proportion of patients who were not up-to-date with their viral load test was high. Six facilities displayed significantly higher, and three facilities significantly lower than average proportions of failure on indicator 2. Again, with four facilities showing a proportion of failure close to 100%, our results suggest that some sites faced systematic barriers for implementing viral load testing guidelines, such as lack of training, lack of lab supplies, lack of sample transport capability, or lack of integration of the sites within the national reference laboratory network. Lastly, we see on Fig. 1c that three facilities displayed a significantly higher than average proportion of failure to use MMS appropriately, while four facilities displayed a significantly lower than average proportion of failure.

### Continuity of care indicators

Figure 2 shows funnel plots for each continuity of care indicators. Figure 2a shows that four facilities displayed higher than expected rates of patients failing to pick up their medication within 30 days of refill date. Six facilities displayed significantly higher, and three facilities significantly lower than expected rates of non-pregnant patients failing to be retained on ART 6 months after initiation (Fig. 2b). 6-month retention among pregnant and postpartum patients at ART initiation showed little evidence of potential outlying performance, with only two low performers and one high performer. Indicator 4 showed limited evidence of over-dispersion, while indicators 5 and 6 showed no evidence of over-dispersion (hence, the overlap of the solid and dashed lines on Fig. 2b and c). This suggests that our case-mix adjustment successfully accounted for most of the differences in patients’ characteristics between facilities. Regarding indicator 4, over-dispersion was expected: timely ART pick-up constitutes a repeated measure, as most patients had several prescriptions over the study period, and intra-patient correlation constitutes a violation to the assumption of independence between binomial trials, which is likely to engender over-dispersion [30].

### Composite measure of QoC

**Fig. 3:**
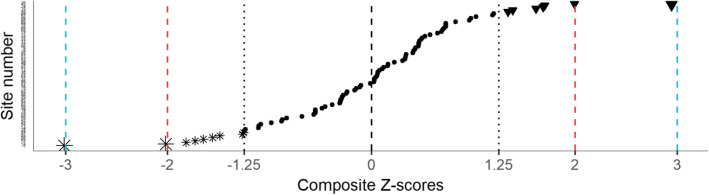
Distribution of the facility-specific composite z-scor. Sites with typical (●), and outlying low (▼) or high (⁎) performances. **±2.00**. **±3.00**

Figure 3 shows the distribution of the composite z-scores. The steps to construct this composite measure of QoC, described above and in Additional file 1, ensured that it behaves as any regular z-score – mean of 0, standard deviation of 1. Therefore, most composite z-scores were expected to lie within one standard deviation from 0. The three facilities outside the ±2 interval show clear evidence of outlying low (Z > 2), and high (Z < − 2) performances. Apart from these three clear-cut facilities, several facilities with composite z-scores significantly different from zero, can be seen as potential outliers.

### Sensitivity analysis

**Fig. 4:**
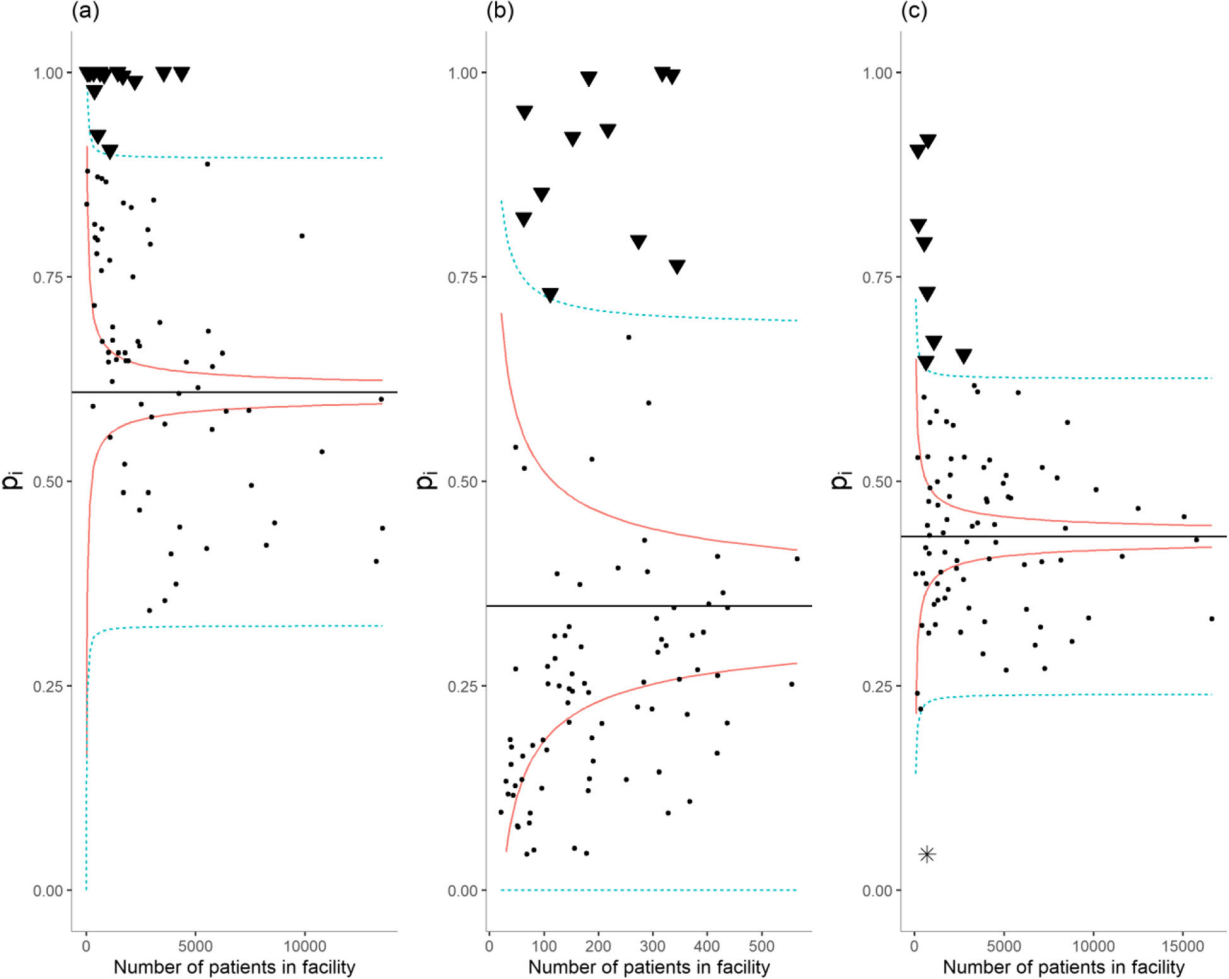
Funnel plots of facility-specific proportion of patients who were not initiated on treatment within the month of their HIV diagnosis (**a**), who were not up-to-date with their viral load test (**b**), who were inappropriately assigned a MMS prescription (**c**), using an additive random-effects model. Sites with typical (●), and outlying low (▼) or high (⁎) performances.  Bonferroni-corrected 95% confidence interval.  Bonferroni-corrected 95% confidence interval accounting for over-dispersion

**Fig. 5:**
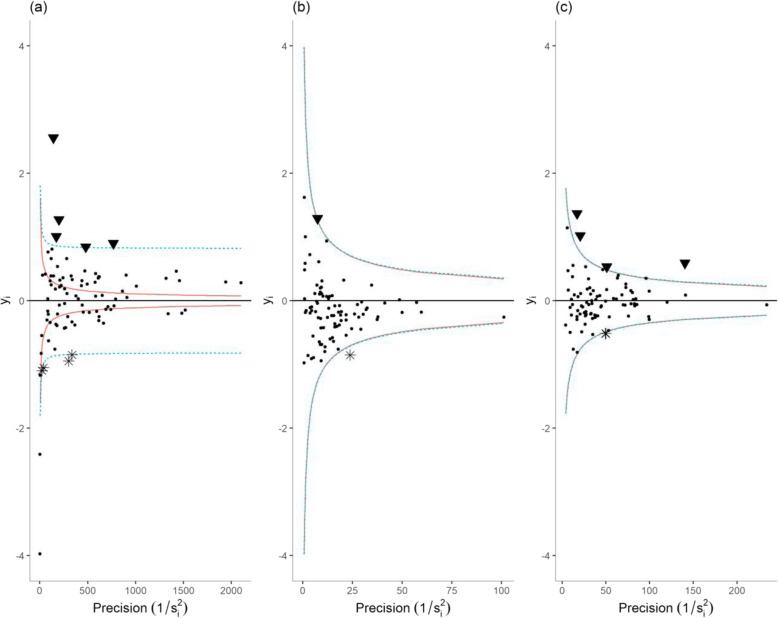
Funnel plots of facility-specific logit ratio of observed versus expected rates of timely ART pick-up (**a**), of 6-month retention among non-pregnant adults (**b**), of 6-month retention among pregnant or post-partum women at ART initiation (**c**), using an additive random-effects model. Sites with typical (●), and outlying low (▼) or high (⁎) performances.  Bonferroni-corrected 95% confidence interval.  Bonferroni-corrected 95% confidence interval accounting for over-dispersion

Our first sensitivity analysis consisted of using an additive random-effects model instead of a multiplicative model to account for over-dispersion. The results appear in Fig. 4-5 (Additional file 1). Although not leading to fundamentally different results, the random-effects approach conduces to identify an inappropriately high number of providers as outliers among facilities with low volume of patients/prescriptions; conversely, it tends to be too lenient with facilities with low volume of patients/prescriptions.

**Fig. 6:**
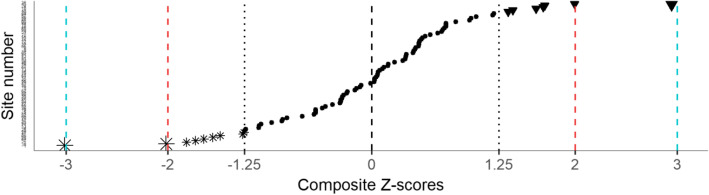
Distribution of the facility-specific composite z-score, not using the relevance weights. Sites with typical (●), and outlying low (▼) or high (⁎) performances.  ±2.00.  ±3.00

Our second sensitivity analysis removed the relevance weights from the calculation of the composite z-score. Figure 6 (Additional file 1) shows the distribution of these composite z-scores. We see that it is almost identical to the distribution in Fig. 3, suggesting that these relevance weights had little impact on the final results.

## Discussion

Our work demonstrates the potential of leveraging existing routine data systems such as i*Santé* along with appropriate statistical methods for institutional performance monitoring. The rich person-level data combined with robust statistical models allowed us to detect evidence of unusual performance among facilities across a series of HIV program indicators. We found that several facilities failed to implement the `test and start’ strategy for most or the totality of their patients, or to monitor HIV viral load according to guidelines, while other facilities performed fairly well on these indicators. Inspections of these facilities could inform whether these disparities reflect variability in the uptake of guidelines, or inequities in access to key resources, such as a steady supply chains of ART or a laboratory performing viral load testing. Additionally, our results indicated a fairly homogeneous uptake of the MMS strategy across facilities, which could reflect the Ministry of Health’s recent efforts to promote this strategy. Our analysis also highlighted significant variation, even after risk adjustment, in facilities’ ability to retain their patients on treatment. The composite measure of process and continuity of care indicators, which reflected an underlying construct of overall QoC, presented several advantages. While processes of care indicators reflect the technical performance of the healthcare providers, continuity of care indicators give a measure of their effects on specific clinical objectives. Based on the composite QoC metric, two facilities displayed clear evidence of outlying high performance and one facility showed signs of outlying poor performance. An in-depth study of these facilities could shed light on the barriers and facilitators for the delivery of appropriate HIV care.

To apply the statistical methods exposed in the provider profiling literature to a real case study, it was necessary to make several analytic choices. First, we chose to weight indicators based on the quality of the data used to construct them and their relevance to quality of care. In a sensitivity analysis, where the six indicators had an equal weight of one, we showed that the relevance weights did not dramatically modify the results. Second, some of our indicators displayed substantial over-dispersion that we estimated and accounted for. In a sensitivity analysis, we considered an additive random-effects model. Although not leading to fundamentally different results, this random-effects approach tended to identify too many providers as outliers, especially for facilities with low volume of patients/prescriptions, a pattern already stressed in previous studies [21]. Lastly, our approach is based on a hypothesis-testing rather than a “model-based” Bayesian hierarchical modeling approach. Arguments in favor of the former over the latter are extensively developed in the discussion section of Spiegelhalter’s seminal work [21]. The main rationale for using a hypothesis-testing framework coupled with *p-values* is that it closely matched the goal of our analysis: detecting divergent facilities. Furthermore, our sensitivity analysis arguably explored the results of using a model-based approach, as the additive random-effects model was essentially an empirical Bayes hierarchical model.

### Limitations

This work is subject to a number of limitations. First, we were restricted by the variables available within i*Santé* to adjust for differences between patients for continuity of care indicators. For example, we had no access to information on patients’ socioeconomic status within the EMR. Thus, our case-mix adjustment might fail to account for potential socio-economic confounders. However, a common symptom of an inadequate case-mix adjustment is high over-dispersion. Out of the three indicators that used a case-mix adjustment, two showed no evidence of over-dispersion, and one displayed little over-dispersion, which suggests that we successfully integrated important predictors in our risk-adjustment model. Second, the funnel plots revealed that facilities with a small number of patients could “get away” with higher scores on our indicators, without being labeled as outliers. While this could be seen as a limitation, we argue that it is the strength of modeling approaches over scorecard evaluation, as it reflects the increased sampling variability expected in smaller units. It is a recognition that we know less about smaller facilities, and therefore need longer observation periods and more data to assess with greater certainty the appropriateness of care in those settings.

Third, indicators of facilities’ resources, such as number of beds or health workers, laboratory capacities or treatment availability are outside the scope of the i*Santé* data, but are pre-conditions of many aspects of appropriate care. For instance, creating and managing the ART supply chain across an entire country can be challenging, and several studies have documented recurrent ART stock outs in LMICs [31–33]. Availability of ART is a pre-condition of two of the three processes of care considered in this study; therefore, the low uptake of the ‘test and start’ and of the MMS strategies observed in certain facilities might be the results of strategies to cope with stock-outs of ART and prevent treatment discontinuation. Fourth, there is an inevitable degree of arbitrariness in the choice of a threshold, which depends on the aim and the resources of the analysis. A country with limited resources and challenges in physical access to certain locations, such as Haiti, may want to focus on a few top outliers (±2). Conversely, working in the context of a richer country, the UK, Bardsley et al. considered a more extensive list of outlying facilities, using ±1.25 as their threshold, which corresponds to the 10% highest and 10% lowest areas under the curve of the theoretical standard normal distribution. Furthermore, the qualitative stakeholder input for weighting the indicators based on data quality and relevance was undertaken with only a small set of individuals. This could be widened and collected in a more structured manner, such as a Delphi process, in the future. Fifth, we chose indicators of HIV QoC based on international metrics, and based on the Haiti HIV National Program’s recommendations. As HIV guidelines and evidence-based care evolve over time, this list of indicators could be modified and enriched. Finally, our analysis rests upon the assumption that i*Santé* data were rigorously and uniformly recorded. Previous assessments of iSanté data quality revealed improvement in i*Santé* data quality over time and generally strong levels of accuracy in data on ART dispensing [12], as well as strong concordance in measures of ART retention derived from a review of paper-based ART registers compared to measures derived from iSanté [34]. While no data quality assessment is available for the period of our analysis, the prior assessments lend credibility to our assumption.

### Future work

We envision several directions for building on this work. First, the results could be leveraged to inform the targeted selection of health facilities for in-depth inspections to learn from star performers and implement corrective measures for poor performers. From an operational standpoint, targeted rather than random inspection of facilities enables to focus resources on sites of greatest interest. This type of targeting could reduce the costs for a national authority regulating the QoC in health institutions. Second, the processing and analysis of the data could be automated as a module layered within the networked EMR. This could provide regular feedback to HIV facilities on how they perform on a set of indicators, and support early detection of facilities with extremely strong or weak performance. Finally, our approach could be replicated in conjunction with other large-scale networked EMRs or other networked health-sector databases containing person-level data about health services, in other areas of primary care, such as antenatal, neonatal, child or maternal care.

## Conclusion

Using methods of health care performance profiling previously applied in resource-rich settings, we constructed a composite measure of QoC within Haiti’s national ART program. This composite measure could be used as part of a continuous quality improvement framework to reduce the disparities in HIV care across Haitian facilities. Furthermore, the analytic approach and modeling choices we used could be generalized to other routine health information systems in low resource settings for healthcare performance monitoring. By demonstrating the potential of statistical methods for health facility performance profiling in one LMIC, we hope our work will act as an incentive to leveraging investments in existing health information systems to inform healthcare quality management, by building local capacity to identify and solve local QoC problems.

## Supplementary information

**Additional file 1.**

## Data Availability

The iSanté data are governed by Haiti’s Ministry of Public Health and Population (MSPP), and permission to access and use the iSanté data must be obtained by contacting the lead for data systems at the Unité de Gestion de Project (UGP): Lavoisier Lamothe, lavoisier.lamothe@ugp.ht

## Abbreviations

HIV: Human immunodeficiency virus
QOC: Quality of care
LMICs: Low and middle-income countries
EMR: Electronic medical records
ART: Anti-retroviral therapy
RHIS: Routine health information system
WHO: World Health Organization
PEPFAR: US President’s Emergency Plan for AIDS Relief
MMS: Multi-month scripting
