# Supplementary material for: Closing the gap in implementation of HIV clinical guidelines in a low resource setting using electronic medical records
Source: BMC Health Serv Res. 2020 Aug 26;20:804. doi: 10.1186/s12913-020-05613-8 (PMC7449080; doi:10.1186/s12913-020-05613-8)
Supplement: Supplementary file 1 — Additional file 1. [file 12913_2020_5613_MOESM1_ESM.docx]

# Appendix 1: Supplemental information

## Calculation of the unadjusted z-scores:

### Processes of care

For each indicator k = 1, 2, 3, the z-score in facility i = 1 ,…, 90, is defined as:

$z_{ik}=n^{1/2}\frac{{(p}_{ik}-\pi_{0k})}{{\pi_{0k}(1-\pi_{0k})}^{1/2}}$ , where $p_{ik}$ is the proportion of failure in facility i on indicator k, and $\pi_{0k}$ the average proportion of failures across facilities on indicator k.

### Outcomes

For each indicator k = 4, 5, 6, the z-score in facility i = 1 ,…, 90, is defined as:

$z_{ik}=\frac{{(y}_{ik}-y_{0k})}{s_{ik}}$ , where $y_{0k}$ is the average value of the indicator k across facilities.

## Estimation of over-dispersion

### Calculation of φ

For indicator k = 1, 2, 3, 4, 5, 6, we estimated φ, the over-dispersion factor, using the following:

$\hat{\varphi_{k}}= \sum_{i=1}^{I} \frac{z_{ik}}{I}$ with I = 90, the total number of facilities considered in the analysis.

We can then conduct a formal test for over-dispersion in the distribution of indicator k (H_0_: no over-dispersion): under H_0_, $I\times\hat{\varphi}_{k}\sim\chi_{I}^{2}$. Therefore, we can calculate a *p-value* for H_0_ by comparing $I\times\hat{\varphi}_{k}$ with the critical value of a chi-distribution for a given threshold for α, the type I error. Using *α = 0.05*, we failed to reject H_0_ for indicators 5 and 6.

For indicators 1, 2, 3, and 4, we rejected H_0_, and thus used $\hat{\varphi}_{k}$ to calculate adjusted z-scores.

### Adjusted z-scores

For indicator k = 1, 2, 3, 4, we adjusted facilities’ z-scores for over-dispersion:

$$z_{ik}^{adjusted}= \frac{z_{ik}}{\hat{\varphi}_{k}}$$

For indicator k = 5, 6, $z_{ik}^{adjusted}= z_{ik}$.

## Calculation of the composite z-score:

1. For each indicator k = 1, 2, 3, 4, 5, 6, we first transformed the z-scores to bring any value beyond ±3 to ±3:

$$\tilde{z}_{ik}=\left( z_{ik},3 \right)\times I\left( z_{ik}>0 \right)+max(z_{ik}, -3)\times I(z_{ik}<0)$$

2. We multiplied each transformed z-score $\tilde{z}_{ik}$ with its relevance weight $\varsigma_{k}$:

$\check{z}_{ik}= \tilde{z}_{ik}\times\varsigma_{k}$, with $\varsigma_{1}=1.5, \varsigma_{2}=1, \varsigma_{3}=1.5, \varsigma_{4}=1.5, \varsigma_{5}=0.5, and \varsigma_{6}=0.5$

1. We then computed a composite z-score, noted $Z_{i}^{composite}$, for each facility i = 1 ,…, 90, using the following formula:

$$Z_{i}^{composite}= \frac{\sum_{k=1}^{k=6} w_{k}\check{z}_{ik}}{\sqrt{\Sigma_{k}\Sigma_{\dot{j}}}w_{k}w_{j}c_{kj}}$$

$where c_{kj} are the correlations between the z-scores and c_{kk}=1,$ and $w_{k}=\frac{1}{\Sigma_{j}c_{kj}}$ to down weight pairs of highly correlated z-scores

1. Finally, we de-meaned and standardized our composite measure:

$\tilde{Z}_{i}^{composite}= \frac{Z_{i}^{composite}- mean(Z_{i}^{composite})}{sd(Z_{i}^{composite})}$ , such that $\tilde{Z}_{i}^{composite}$ behaves as a z-score (mean of 0 and standard deviation of 1)

We calculated it for 89 out of the 90 facilities, as one facility had no patient eligible for three out of the six indicators.

# Appendix 2: Supplemental Figures

## An additive random-effects model

Alternatively to the multiplicative over-dispersion model, we used an additive random-effects model that accounts for over-dispersion by adding a parameter τ to the standard error of the null distribution s_0_: τ reflects the unavoidable variability between facilities due to unexplainable factors (also called the *nugget effect* in geospatial statistics).

**Figure 4**

**Figure 5**

## Not using relevance weights in the calculation of the composite z-score

We decided to use relevance weights in the main body of the text, to incorporate qualitative intelligence into quantitative intelligence, and reflect the reliability and relevance of each indicator to overall QoC. As a sensitivity analysis, we present here the unweighted composite z-scores, which corresponds to skipping step 2) in **Appendix 1.3**.

Figure 6
